# Isolation and cloning of large subunit of Influenza virus A (H1N1) hemagglutinin gene into Bacmid vector to construct recombinant Baculovirus

**DOI:** 10.1186/1753-6561-5-S1-P12

**Published:** 2011-01-10

**Authors:** S Hossain Zadeh, F Fotouhi, MT Kheiri, MR Razavi, B Heydarchi, B Farahmand, S Najafi

**Affiliations:** 1 Department of Cellular and Molecular Biology, Faculty of Biological Sciences, Science and Research branch of Islamic Azad University, Tehran, Iran; 2 Influenza unit, Department of Virology, Pasteur Institute of Iran, Tehran, Iran; 3 Department of Mycobacteriology & Pulmonary Research, Pasteur Institute of Iran, Tehran, Iran

## 

Influenza virus A (H1N1) is an important subtype of influenza virus that makes numerous consequences throughout the world. In the first step of viral attachment, main antigenic site of the HA1 domain from the globular head of hemagglutinin (HA) binds to human cell receptors, starting the disease process. In order to produce recombinant subunit protein vaccines, we focus on the nucleotide sequence of HA1 gene to generate the recombinant baculovirus shuttle vector (bacmid) to produce recombinant baculovirus in sf9 insect cells.

For this purpose, the human influenza virus A /New Caledonia 20/1999/ (H1N1) was propagated in MDCK cell culture and viral RNA was extracted using Easy-red (iNtRON) solution. Complementary DNA synthesis and HA1 amplification was carried out using uni-12 primer and HA1 specific primers respectively. Expected PCR product was evaluated through 1% agarose gel, confirmed by restriction enzyme analysis, cloned into pGEM-TEasy vector (Promega) and completely sequenced. The gene of interest was digested from cloning T-vector and subcloned into pfastBac HT donor plasmid, confirmed by PCR, digestion and sequencing. The recombinant donor plasmid was transformed into the E.coli DH10Bac competent cells for site-specific transposition of the HA1 from the donor plasmid to a bacmid DNA through lacZ gene disruption. The high-molecular-weight bacmid DNA was isolated from the overnight cultures and verified by electrophoresis on 0.5% agarose gel and PCR analysis using either M13/pUC or gene specific primers. We are going to transfect sf9 insect cells with this recombinant Bacmid to generate recombinant baculovirus and produce large amount of HA1 protein for future studies. This is the first study of recombinant HA1 production in eukaryotic system in Iran.

